# Severe COVID‐19 correlates with lower mitochondrial cristae density in PBMCs and greater sitting time in humans

**DOI:** 10.14814/phy2.15369

**Published:** 2022-07-26

**Authors:** Mauricio Castro‐Sepulveda, German Tapia, Mauro Tuñón‐Suárez, Alejandra Diaz, Hugo Marambio, Mayalen Valero‐Breton, Rodrigo Fernández‐Verdejo, Hermann Zbinden‐Foncea

**Affiliations:** ^1^ Exercise Physiology and Metabolism Laboratory (LABFEM), School of Kinesiology Faculty of Medicine, Finis Terrae University Santiago Chile; ^2^ Sports Health Center Santa María Clinic Santiago Chile; ^3^ Institute of Neuroscience, UCLouvain Louvain‐La Neuve Belgium

**Keywords:** COVID‐19, metabolism, mitochondrial dynamics, mitochondrial fusion, PBMCs, SARS‐CoV‐2

## Abstract

An interaction between mitochondrial dynamics, physical activity levels, and COVID‐19 severity has been previously hypothesized. However, this has not been tested. We aimed to compare mitochondrial morphology and cristae density of PBMCs between subjects with non‐severe COVID‐19, subjects with severe COVID‐19, and healthy controls. Additionally, we compared the level of moderate‐vigorous physical activity (MVPA) and sitting time between groups. Blood samples were taken to obtain PBMCs. Mitochondrial dynamics were assessed by electron microscopy images and western blot of protein that regulate mitochondrial dynamics. The International Physical Activity Questionnaire (IPAQ; short version) was used to estimate the level of MVPA and the sitting time The patients who develop severe COVID‐19 (COVID‐19++) not present alterations of mitochondrial size neither mitochondrial density in comparison to non‐severe patients COVID‐19 (COVID‐19) and control subjects (CTRL). However, compared to CTRL, COVID‐19 and COVID‐19++ groups have lower mitochondrial cristae length, a higher proportion of abnormal mitochondrial cristae. The COVID‐19++ group has lower number (trend) and length of mitochondrial cristae in comparison to COVID‐19 group. COVID‐19, but not COVID‐19++ group had lower Opa 1, Mfn 2 and SDHB (Complex II) proteins than CTRL group. Besides, COVID‐19++ group has a higher time sitting. Our results show that low mitochondrial cristae density, potentially due to physical inactivity, is associated with COVID‐19 severity.

## Graphical abstract

The COVID‐19++ group (severe COVID‐19) have lower length of mitochondrial cristae in comparisons to COVID‐19 group (non‐severe COVID‐19). The COVID‐19++ group have a higher time sitting in comparisons to COVID‐19 group.

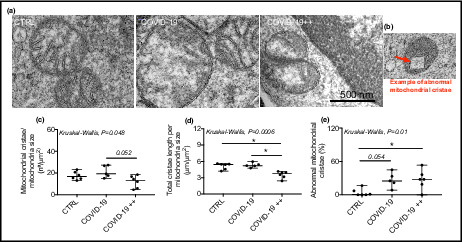

## 1 INTRODUCTION

The coronavirus disease 2019 (COVID‐19), caused by severe acute respiratory syndrome coronavirus 2 (SARS‐CoV‐2), has triggered one of the worst recent pandemics. To fight SARS‐CoV‐2 infection, the immune system of the host releases several cytokines. For example, patients with COVID‐19 have been shown to have ~8‐fold higher plasma concentration of interleukin‐6 than healthy volunteers (Ajaz et al., 2021). Unfortunately, this protective response may result in multiorgan failure in cases of severe COVID‐19 (Mangalmurti & Hunter, 2020). The search of risk factors of severe COVID‐19 has thus become essential to protect vulnerable subjects. Early data identified age, obesity, and underlying comorbidities as major risk factors of severe COVID‐19 (Wang et al., 2020; Zhu et al., 2020). A low cardiorespiratory fitness –partially explained by physical inactivity– was initially suggested to also play a role (Castro‐Sepúlveda et al., 2021; Zbinden‐Foncea et al., 2020), and this was subsequently confirmed (Brawner et al., 2021). The underlying mechanisms that link these factors with severe COVID‐19 are, however, unknown.

Peripheral Blood Mononuclear Cells (PBMC) are critical blood cells that help the immune system function properly. Circulating monocytes enter tissues, and then become macrophages able to phagocytize viruses. Notably, the phagocytic capacity of macrophages is proposed to be largely dependent on mitochondria (Tur et al., 2017). Mitochondrial function may therefore be essential to face SARS‐CoV‐2 infection, and thus determine COVID‐19 severity (Burtscher, Millet, & Burtscher, 2021; Burtscher, Burtscher, & Millet, 2021; Castro‐Sepúlveda et al., 2021). In agreement, COVID‐19 patients show signs of mitochondrial dysfunction in PBMCs (Ajaz et al., 2021). Specifically, their PBMCs had lower ATP‐linked respiration, maximal respiration, and reserve capacity than PMBCs from healthy volunteers (Ajaz et al., 2021). Perturbations in mitochondrial dynamics, including mitochondrial size (mitochondrial fusion) and density, as well as mitochondrial cristae density, could also be involved. Indeed, perturbed mitochondrial dynamics (fusion/fission) are a feature of populations at high risk of severe COVID‐19, including the elderly (Sebastián et al., 2016), subjects with type 2 diabetes (Kelley et al., 2002), and subjects with low cardiorespiratory fitness (Castro‐Sepulveda et al., 2020; Castro‐Sepulveda et al., 2021; Nielsen et al., 2017).

We thus hypothesized that elevated COVID‐19 severity associates with perturbations in the mitochondrial dynamics of PBMCs and lower physical activity. We aimed to compare mitochondrial morphology, size and cristae density of PBMCs, and between subjects with non‐severe COVID‐19, subjects with severe COVID‐19, and healthy controls. Besides, we compared the level of moderate‐vigorous physical activity (MVPA) and sitting time between groups.

## 2 METHODS

### 2.1 Design and participants

This was an observational cross‐sectional study. We recruited men with diagnosed COVID‐19 (by RT‐PCR) and healthy subjects (pathological control; CTRL) who met the following criteria: [a] 45 to 65 years old; [b] excess body weight ([excess body weight patients are more likely to severe COVID‐19] body mass index between 25 and 35 kg/m^2^); and [c] without diagnostic of diabetes or hypertension. At their arrival to the clinic (from January to May 2021), blood samples (~10 ml) were drawn from all subjects to isolate PBMCs. Among patients, those with non‐severe COVID‐19 were sent home to spend a quarantine (COVID‐19 group), whereas those with severe COVID‐19 were admitted to the intensive care unit for ventilatory support (COVID‐19++ group). Hospitalization criteria were: [a] need for ventilatory support or oxygen therapy to maintain oxygen saturation; or [b] partial or complete respiratory failure. COVID‐19 patients personally answered the International Physical Activity Questionnaire (IPAQ). COVID‐19++ subjects did so, after emergency procedures, once clinically stabilized; and COVID‐19 subjects on the day of blood collection. Written informed consent was obtained from participants, and all the procedures were approved by the Research Ethics Committee of Santa Maria Clinic.

### 2.2 PBMCs isolation

PBMCs were separated from blood under sterile conditions on a Ficoll‐Histopaque 1077 (Sigma, Milan, Italy), as previously described (Castro‐Sepúlveda et al., 2021). Part of PBMCs was frozen in liquid nitrogen and stored at −80°C for western blotting; while the remaining was fixed in 2.5% glutaraldehyde for transmission electron microscopy.

### 2.3 Western blotting

PBMCs protein extracts were denaturalized at 95 °C for 5 min in Laemmli buffer. Proteins were separated by SDS‐PAGE, and transferred to PVDF membranes, as previously described (Castro‐Sepúlveda et al., 2021). The following antibodies and dilutions were used: Mfn 2 (Mfn 2, ab56889, Abcam; 1:1000), Optic atrophy 1 (Opa 1, 612,606, BD Biosciences; 1:1000) and total Oxidative phosphorylation cocktail (OXPHOS, ab110413, Abcam; 1:1000). Complex IV was used as loading control of mitochondrial mass (Castro‐Sepúlveda et al., 2021). Under proteins denaturation conditions (95°C for 5 min) not all complex‐subunits of the OXPHOS antibody are detected.

### 2.4 Transmission electron microscopy

PBMCs were prepared as previously described (Castro‐Sepúlveda et al., 2021). Sections of 80‐nm were cut, mounted on electron microscopy grids, and examined using a transmission electron microscope (Philips, Tecnai 12 at 80 kV). Mitochondrial density was evaluated as previously described (Castro‐Sepulveda et al., 2021; Castro‐Sepúlveda et al., 2021; Echeverria et al., 2021). Mitochondrial density (%) and size (μm^2^) were assessed in 4–6 cells per subject, and 3–10 mitochondria per cells. Mitochondrial cristae density (cristae number and cristae length) was assessed as previously described (Castro‐Sepulveda et al., 2021; Echeverria et al., 2021). The mean of mitochondrial size and density, and cristae density was calculated for each subject and used in statistical analyses.

### 2.5 IPAQ

The IPAQ (short version) was used to estimate the level of moderate‐vigorous physical activity (MVPA) and the sitting time (World Health Organization, n.d.; Craig et al., 2003). Patients responded the IPAQ considering the week before the beginning of the pandemic. For calculations, vigorous activities are assumed to require 8 metabolic equivalents (MET), moderate activities 4 MET, and walking 3.3 MET. Note that 3.3 MET classify as a moderate‐intensity activity according to current cutoffs (Bull et al., 2020). The level of MVPA was expressed in MET×min/wk, as previously done (Fernández‐Verdejo & Suárez‐Reyes, 2021). Subjects were classified as active if achieving ≥600 MET×min/wk, or inactive if not (Bull et al., 2020; Fernández‐Verdejo & Suárez‐Reyes, 2021). The IPAQ also collects information about the daily sitting time, which was expressed in min/d.

### 2.6 Statistics

Data are presented as median and interquartile range. Kruskal‐Wallis with the Dunn's post‐hoc was used to compare continuous variables between groups (CTRL, COVID‐19 and COVID‐19++). Mann–Whitney U was used to compare MVPA and sitting time between COVID‐19 and COVID‐19++. Fisher's exact test was used to compare the proportion of symptoms, signs, the number of physically active subjects between the COVID‐19 and COVID‐19++ group. Association between mitochondrial cristae density markers and sitting time was testing by Spearman test. *P* < 0.05 was considered as statistically significant and *P* < 0.09 was considered trend. Prism 7 (GraphPad Software, La Jolla, CA, USA) was used for analyses.

## 3 RESULTS

### 3.1 General characteristics of the subjects

Groups had no difference in age (CTRL, 50.0 [46.8–55]; COVID‐19, 48.0 [41.0–55.5]; COVID‐19++, 49.5 [46.8–54.3]; *P* = 0.80), height (CTRL, 1.71 [1.67–1.73]; COVID‐19, 1.68 [1.61–1.80]; COVID‐19++, 1.73 [1.69–1.82]; *P* = 0.68), weight (CTRL, 82.6 [76.1–88.3]; COVID‐19, 75.0 (68.0–87.0); COVID‐19++, 86.5 [79.0–97.8]; *P* = 0.34), and body mass index (CTRL, 29.3 [26.9–30.]); COVID‐19, 26.2 [25.5–27.3]; COVID‐19++, 28.9 [25.9–31.2]; *P* = 0.21). Moreover, COVID‐19 and COVID‐19++ groups had no difference in the prevalence of signs and symptoms (fever: COVID‐19 [100%]; COVID‐19++ [83%]; *P* = 0.99, cough: COVID‐19 [20%]; COVID‐19++ [33%]; *P* = 0.99, myalgia: [20%]; COVID‐19++ [17%]; *P* = 0.99) and dyspnea: [80%]; COVID‐19++ [100%]; *P* = 0.45).

### 3.2 Mitochondrial morphology

**FIGURE 1:**
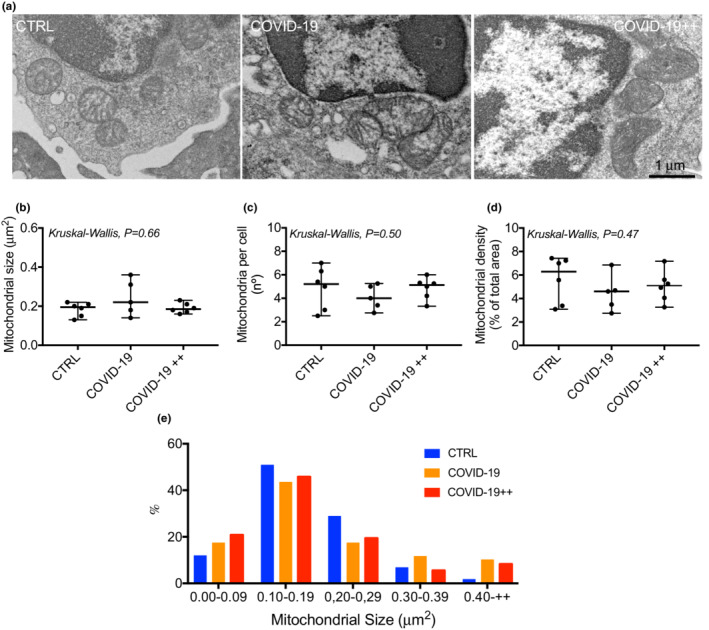
Mitochondrial morphology in PBMCs. (a) Representative transmission electron microscopy images of mitochondrial morphology in human PBMCs of control healthy subjects (CTRL), patients with non‐severe COVID‐19 (COVID‐19), and patients with severe COVID‐19 (COVID‐19++). (b–d) quantification of mitochondrial (b) size, (c) number, and (d) density. (e) Mitochondrial size distributions. *N* = 5–6 per group; each point represents the mean of 4–6 cells and of >5 mitochondria/cell for each subject. Data are presented as median, minimum, and maximum. Kruskal‐Wallis was used to compare groups.

Figure 1a shows representative images of the mitochondrial morphology in PBMCs of each group. Groups had neither difference in mitochondrial size (Figure 1b,e), number (Figure 1c), nor density (Figure 1d) (*P* > 0.05).

**FIGURE 2:**
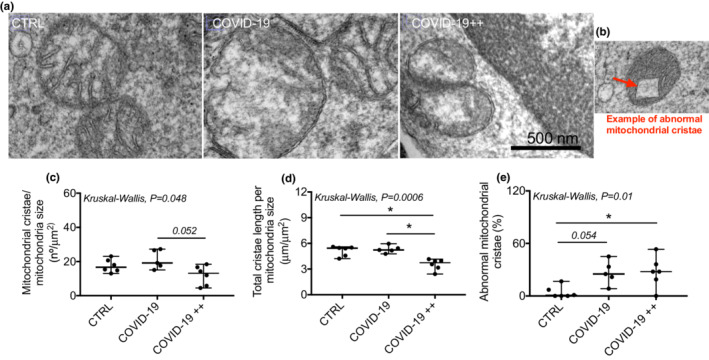
Mitochondrial cristae density and morphology in PBMCs. (a) Representative transmission electron microscopy images of mitochondrial morphology in human PBMCs of control healthy subjects (CTRL), patients with non‐severe COVID‐19 (COVID‐19), and patients with severe COVID‐19 (COVID‐19++). (b) Example of an abnormal mitochondrial cristae. (c, e) quantification of mitochondrial cristae (c) size, and (d) length. (e) Percentage of abnormal cristae. *N* = 5–6 per group; each point represents the mean of 4–6 cells and of >5 mitochondria/cell for each subject. Data are presented as median, minimum, and maximum. *P < 0.05 in the post‐hoc test. Kruskal‐Wallis with the Dunn's post‐hoc was used to compare groups.

Figure 2a shows representative images of mitochondrial cristae in PBMCs of the groups, and Figure 2b examples of abnormal mitochondrial cristae. COVID‐19++ group had (*P* = 0.052) lower (trend) mitochondrial cristae number than COVID‐19 (Figure 2c; *P* = 0.052). COVID‐19++ group also had lower cristae length than CTRL and COVID‐19 (Figure 2d). Finally, COVID‐19++ and COVID‐19 (trend) groups had a higher percentage of abnormal mitochondrial cristae than CTRL (Figure 2e).

### 3.3 Mitochondrial fusion and OXPHOS proteins

**FIGURE 3:**
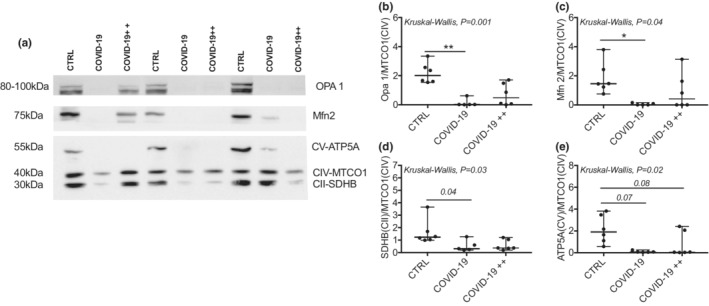
Mitochondrial protein content in in PBMCs. (a) Representative blots in control healthy subjects (CTRL), patients with non‐severe COVID‐19 (COVID‐19), and patients with severe COVID‐19 (COVID‐19++). (b–e) quantification of the protein content of (b) Opa 1, (c) Mfn 2, (d) SDHB (complex II), and (e) ATP5A (complex V). *N* = 5–6 subjects per group. Data are presented as median, minimum, and maximum. *P < 0.05 and **P < 0.01 in the post‐hoc test. Kruskal‐Wallis with the Dunn's post‐hoc was used to compare groups.

Figure 3a shows representative images of the mitochondrial fusion and OXPHOS proteins content in each group. We found no differences in MTCO1 protein (Complex IV) protein between groups when adjusted to Red Ponceau *P = 0.80* (data not shown). Therefore, we use MTCO1 protein (Complex IV) as a mitochondrial load control. COVID‐19, but not COVID‐19++ group, had lower Opa 1 and Mfn 2 than CTRL group (Figure 3b,c). With respect to OXPHOS proteins, the SDHB (Complex II) also is lower in COVID‐19, but not COVID‐19++ group (Figures 3d) in comparison to CTRL group. The ATP5A (Complex V) protein was lower (trend) in both, COVID‐19 and COVID‐19++ groups (Figures 3e) than CTRL group. Similar results were found when mitochondrial proteins were adjusted to Red Ponceau (data not shown).

### 3.4 MVPA and sitting time

**FIGURE 4:**
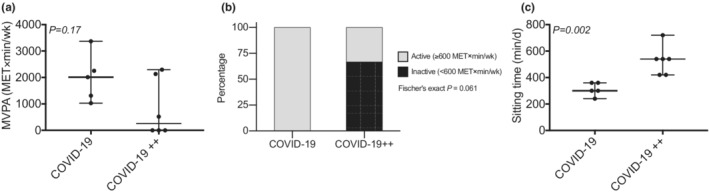
Moderate‐vigorous physical activity (MVPA) and sitting time. (a, c) comparison of the (a) level of MVPA, (b) proportion of physically active and inactive subjects, and (c) sitting time between patients with non‐severe (COVID‐19) and severe (COVID‐19++) COVID‐19. *N* = 5–6 subjects per group. Data are presented as median, minimum, and maximum, or percentages. Mann–Whitney U was used to compare MVPA and sitting time between groups. Fisher's exact test was used to compare the number of physically active subjects between the COVID‐19 and COVID‐19++ group.

COVID‐19 and COVID‐19++ groups had no difference in the level of MVPA (Figure 4a; *P* > 0.05). Yet, COVID‐19++ group had lower prevalence of physically active subjects than the COVID‐19 group (trend, Figure 4b). The sitting time was higher in COVID‐19++ group than COVID‐19 (Figure 4c). Finally, association between sitting time and mitochondrial cristae density markers were found (sitting time v.s. mitochondrial criste number, *r* = −0.75, *P = 0.01*; mitochondrial criste length, *r* = −0.87, *P = 0.001*; abnormal mitochondrial criste, *r* = −0.27, *P = 0.42*).

## 4 DISCUSSION

Here, we investigated the mitochondrial morphology and mitochondrial cristae (density [number and length] and abnormalities) in PBMCs of patients with two grades of severity of COVID‐19: [a] non‐severe, who spent a quarantine at home (COVID‐19); and [b] severe, who entered the intensive care unit (COVID‐19++). We found that, compared to healthy control subjects (CTRL), COVID‐19 patients (COVID‐19 and COVID‐19++) had higher proportion of abnormal mitochondrial cristae. Focusing on severity, COVID‐19++ had lower number and length of mitochondrial cristae (surrogate marker of mitochondrial density), and higher prevalence of sitting time than COVID‐19. These results show that disturbances in mitochondrial cristae –potentially due to low overall levels physical activity– is associated with the risk for severe COVID‐19.

Mitochondrial size is used as an indicator of fusion (larger size) phenotypes (Castro‐Sepulveda et al., 2020; Huertas et al., 2019). Previous measurements of mitochondrial morphology in monocytes from patients with COVID‐19 showed larger mitochondria compared to healthy subjects (Gibellini et al., 2020). Therein, the authors hypothesized that monocytes from COVID‐19 patients accumulated dysfunctional larger mitochondria (Gibellini et al., 2020). Our current results showed no difference in the mean of mitochondrial size between patients with COVID‐19 and healthy controls. However, in the frequency distributions of mitochondrial size, both COVID‐19 and COVID‐19++ groups showed a major percentage of mitochondrial size (+0.40 μm^2^) in comparison to the control group. The potential discrepancy may be explained by differences in the underlying pathologies and age of the patients. The previous study included subjects aged –on average– 63 years old (Gibellini et al., 2020), while ours were 50 years old. Additional studies are thus required whether COVID‐19 patients have larger mitochondria, and whether this associates with the severity of the disease.

Mitochondrial cristae density is a dynamic process that regulates OXPHOS activity (Castro‐Sepúlveda et al., 2021; Echeverria et al., 2021; Yang et al., 2015). A recent study showed that mitochondria of COVID‐19 patients had lower OXPHOS activity compared to healthy subjects and to patients with chest infection (Ajaz et al., 2021). In agreement, herein we found lower content of mitochondrial OXPHOS proteins in patients with COVID‐19, specifically the SDHB (Complex II) and ATP5A (Complex V). Additionally, we studied mitochondrial cristae density and the presence of abnormal mitochondria in patients with COVID‐19. Our results showed, for the first time, that COVID‐19++ patients have lower mitochondrial cristae density. Also, both COVID‐19 and COVID‐19++ groups showed increased abnormal mitochondrial cristae. We speculate that the decreased OXPHOS activity previously observed in COVID‐19 patients (Ajaz et al., 2021) could be due to a reduced mitochondrial cristae density. Mitochondrial cristae density may be also associated with the severity of COVID‐19. Indeed, our COVID‐19++ group showed lower mitochondrial cristae density than the COVID‐19 group. To gain insight into possible mechanisms, we measured the content of proteins that regulate mitochondrial cristae density (Castro‐Sepulveda et al., 2021; Sood et al., 2014). Both OPA 1 and Mfn2 were lower in patients with COVID‐19 compared to healthy subjects, yet no differences between COVID‐19 groups were apparent. The dramatic decrease in mitochondrial proteins in COVID‐19 patients (e.g., Opa 1), without mitochondrial morphological nor mitochondrial density alterations, must be further confirmed and analyzed. Therefore, none of these proteins appear to play a role in the severity of COVID‐19. Future studies should measure other proteins that also regulate mitochondrial cristae density, such as MICOS complex like MIC60 (Castro‐Sepulveda et al., 2021; Yang et al., 2015).

During hospitalization, patients with well‐controlled glycemia show lower mortality than patients with poorly controlled glycemia (upper limit of glycemic variability exceeding 10.0 mmol/L) (Zhu et al., 2020). Also, elevated glucose levels favor SARS‐CoV‐2 infection and monocyte response (Codo et al., 2020). Notably, we have previously shown that exposure of PMBCs in vitro to high glucose decreases mitochondrial cristae density (Castro‐Sepúlveda et al., 2021). Perhaps, high glycemia in patients with COVID‐19 decreases mitochondrial cristae density, thus making them more susceptible to severe COVID‐19. In our current study, unfortunately, we did not measure glycemia, for not having fasting plasma in the COVID‐19 groups. Future studies are thus required to look for a link between glycemia, PMBCs cristae, and COVID‐19 severity. Individuals with low cardiorespiratory fitness also have elevated risk of severe COVID‐19 (Brawner et al., 2021; Sallis et al., 2021). We and others have found that low mitochondrial cristae density in skeletal muscle is associated with reduced cardiorespiratory fitness (Castro‐Sepulveda et al., 2021; Nielsen et al., 2017). We speculate that the protective effects of overall physical activity on COVID‐19 severity are explained by an increased mitochondrial cristae density. This is supported by our data, wherein the COVID‐19 group had more physically active subjects (trend) and less sitting time, along with higher cristae density compared to the COVID‐19++ group. Notably, MVPA and sedentary behavior (i.e., sitting time) may be independent risk factors of severe COVID‐19, as they are already –for example– for all‐cause mortality (Ekelund et al., 2019). Future studies should test that hypothesis.

Our study has certain limitations. First, the small sample size may have precluded us from detecting some differences between groups. Second, the study design does not allow to distinguish whether the differences observed are a cause or consequence of COVID‐19 and/or its severity. Third, the physical activity levels of the CTRL group were not evaluated, and this may be a confounding factor. Finally, another limitation of our study is that no mitochondrial fission factor protein levels were evaluated due to the limited amount of sample of PBMCs. In conclusion, our results show that mitochondrial cristae density, potentially due to physical inactivity, is associated with COVID‐19 severity. However, more studies are needed to confirm causality.

## AUTHOR CONTRIBUTIONS

M.C.S. and H.Z.F conceived and designed research; M.C.S., A.D, H.M, M.T.S., G.T., S.U.C., R.F.C. and M.V.B. performed experiments, analyzed data and interpreted results and M.C.S. R.F.C and H.Z.F drafted manuscript; all authors approved final version of manuscript.

## FUNDING INFORMATION

This study was funded by Funds of Research from Santa Maria Clinic, Santiago, Chile.

## CONFLICTS OF INTEREST

The authors declare no competing interests.
